# Low Rates of Antimicrobial-Resistant Enterobacteriaceae in Wildlife in Taï National Park, Côte d’Ivoire, Surrounded by Villages with High Prevalence of Multiresistant ESBL-Producing *Escherichia coli* in People and Domestic Animals

**DOI:** 10.1371/journal.pone.0113548

**Published:** 2014-12-04

**Authors:** Katerina Albrechtova, Ivo Papousek, Helene De Nys, Maude Pauly, Etile Anoh, Arsene Mossoun, Monika Dolejska, Martina Masarikova, Sonya Metzger, Emmanuel Couacy-Hymann, Chantal Akoua-Koffi, Roman M. Wittig, Jiri Klimes, Alois Cizek, Fabian H. Leendertz, Ivan Literak

**Affiliations:** 1 Department of Biology and Wildlife Diseases, Faculty of Veterinary Hygiene and Ecology, University of Veterinary and Pharmaceutical Sciences, Brno, Czech Republic; 2 Project Group “Epidemiology of Highly Pathogenic Microorganisms”, Robert Koch Institute, Berlin, Germany; 3 Department of Primatology, Max-Planck-Institute for Evolutionary Anthropology, Leipzig, Germany; 4 Research Center for Development -Alassane Ouattara University, University Teaching Hospital Bouaké, Bouaké, Côte d’Ivoire; 5 LANADA, Laboratoire Nationale de la Pathologie Animale, Bingerville, Côte d’Ivoire; 6 CEITEC VFU, University of Veterinary and Pharmaceutical Sciences, Brno, Czech Republic; 7 Institute of Microbiology and Infectious Diseases, Faculty of Veterinary Medicine, University of Veterinary and Pharmaceutical Sciences, Brno, Czech Republic; 8 Centre Suisse de Recherches Scientifiques, Abidjan, Côte d’Ivoire; The Ohio State University, United States of America

## Abstract

Antimicrobial resistance genes can be found in all ecosystems, including those where antibiotic selective pressure has never been exerted. We investigated resistance genes in a collection of faecal samples of wildlife (non-human primates, mice), people and domestic animals (dogs, cats) in Côte d’Ivoire; in the chimpanzee research area of Taï National Park (TNP) and adjacent villages. Single bacteria isolates were collected from antibiotic-containing agar plates and subjected to molecular analysis to detect Enterobacteriaceae isolates with plasmid-mediated genes of extended-spectrum beta-lactamases (ESBLs) and plasmid-mediated quinolone resistance (PMQR). While the prevalence of ESBL-producing *E. coli* in the villages was 27% in people (n = 77) and 32% in dogs (n = 38), no ESBL-producer was found in wildlife of TNP (n = 75). PMQR genes, mainly represented by *qnrS1*, were also present in human- and dog-originating isolates from the villages (36% and 42% in people and dogs, respectively), but no *qnrS* has been found in the park. In TNP, different variants of *qnrB* were detected in *Citrobacter freundii* isolates originating non-human primates and mice. In conclusion, ESBL and PMQR genes frequently found in humans and domestic animals in the villages were rather exceptional in wildlife living in the protected area. Although people enter the park, the strict biosecurity levels they are obliged to follow probably impede transmission of bacteria between them and wildlife.

## Introduction

Antimicrobial resistance (AMR) is a serious problem that affects the dynamics of microbial populations worldwide, pathogens as well as commensals [1]. Evolution of AMR is swift, owing to plethora of mobile genetic elements, short generation periods and adaptability inherent to many bacterial species [2]. It is presumed that the majority of the resistance gene pool co-evolves with the emergence of natural antibiotic traits. However, certain resistance alleles are given advantage by the use of antibiotics in medicine and agriculture [3]. Unfortunately, it is difficult to estimate which alleles of the naturally evolving resistome will become clinically relevant in future [4]. While some AMR genes have been dispersed globally, owing to successful bacterial clones or conjugative plasmids, other are typically found in particular continents, localities or hospital wards. The public health risk of resistance genes varies according to their genetic vehicle (plasmid, transposon, adjacent insertion sequence etc.) and bacterial species carrying the genes [5]. Monitoring of resistance genes occurring at the interface of pristine and human-influenced ecosystems helps us to estimate the frequency of gene exchange in microbial populations of people, domestic animals and wildlife and thereby to better understand the AMR epidemiology.

Extended-spectrum beta-lactamases (ESBL) confer resistance to wide spectrum of beta-lactams, up to third-generation cephalosporins [6]. Their increasing occurrence and diversity, documented since the 1980s [7]–[8], is intertwined with the use of these drugs in human and veterinary medicine. ESBL genes are often transferred on multiresistant conjugative plasmids, which facilitated their global emergence; these are not limited to hospitals but also abundant in community settings [8]. One of the most promiscuous and most widespread among the ESBLs is the CTX-M family [9]. AmpC-type beta-lactamases occur as inherent chromosome-mediated enzymes in several classes of non-pathogenic bacteria and they are being detected with an increasing frequency as plasmid-encoded enzymes in clinically relevant Enterobacteriaceae [10]. Plasmid-mediated quinolone resistance (PMQR) is represented by a heterogeneous group of genes *qnr, aac(6*′*)-Ib-cr, oqxAB* and *qepA*. Different types of genes *qnr* and the *cr-*mutation of *aac(6′)-Ib* gene have low ability to inactivate quinolones and therefore their presence usually does not generate resistance above the clinical breakpoints. Still, these genes play an important role in the multifactorial process of fluoroquinolone resistance generation and monitoring of their prevalence is important to our understanding to fluoroquinolone resistance epidemiology [11]. Although different alleles of *qnr* (specifically *qnrB* and *qnrS*) occur naturally as a part of intrinsic resistance in *Citrobacter* sp. and *Vibrio* sp., their mobilisation to *E. coli* is supposedly a human-influenced phenomenon, driven mainly by the use of fluoroquinolones in human and animal medicine and agriculture [11]. The global increase of Enterobacteriaceae with plasmid-mediated genes *aac(6′)-Ib-cr, oqxAB* and *qepA* is also considered a consequence of quinolone use [12].

ESBLs, AmpCs and PMQR represent a serious public health problem in Africa, where the pressure of infectious diseases is high and the use of antibiotics is often inappropriate [13]. Many studies have investigated resistance and its genetic background in clinical or agricultural settings, but less data have been collected in ecosystems where the human impact is limited to minimum, e.g. nature reserves and other protected areas [14]–[16]. Taï National Park (TNP) in Côte d’Ivoire (CI) is one of the last remaining forest blocks in west Africa, its protection was established in 1970s to conserve endangered population of the western chimpanzee (*Pan troglodytes verus*), the pygmy hippopotamus (*Choeropsis liberiensis*) and other wildlife. Access of people is limited to researchers and local assistants in the northern part of the park. Nevertheless, even with such level of protection, microbial exchange between people and wildlife is not impossible, as demonstrated by the transmission of human respiratory viruses to the chimpanzees [17]–[18].

In this study we aimed to identify the spectrum of resistance genes in gastrointestinal flora of primates living in Taï National Park and inhabitants of villages surrounding the protected area. We were looking at possible overlaps in the spectrum of resistant bacteria in these two ecosystems, adjacent one to the other but totally different in terms of human impact. We address the prevalence of resistant bacteria in NHPs in TNP, as well as people and domestic animals living in its proximity. When discussing these issues, the presence of ESBL-producing Enterobacteriaceae is considered as a result of human-originating microbial pollution [19]–[20]. Indisputably, part of PMQR genes (*aac-6′-Ib-cr, qepA, oqxAB* and *qnr* in *E.* coli) can also be regarded as human-originating pollution, yet another part (*qnrB* in *Citrobacter* sp. or sole *oqxA* in *Klebsiella* sp.) probably represent evolving intrinsic resistance. [21]–[22]. The latter group of PMQR is noteworthy for their potential of transfer to human pathogens.

## Materials and Methods

### Study area

TNP, located at the western border of Côte d’Ivoire (geographic coordinates of the main research camp: lat.5.86767554/long.–7.33968803) represents the largest remaining tropical rainforest in West Africa (435,000 ha) [23]. It is covered with dense primary forest and lined by less dense secondary forest at the borders. Three groups of habituated chimpanzees dwell in the park; northern group (18 individuals in 2012), southern group (24 individuals) and eastern group (31 individuals). Additionally, unhabituated chimpanzees have been observed in the park; their total number is not known. While overlaps of ranging areas of the three groups are minimal and chimpanzees from different groups rarely meet face to face, immigration of females from neighbouring groups can occur. In 2004, 2007 and 2011, the chimpanzees of the South group and in 2007 the individuals of the East group were treated by a single shot of Extencilline (Benzathine Benzylpenicillin 2,400,000 IU; Sanofi-Aventis, France; 3–5 ml intramuscular injection per individual) due to severe respiratory outbreaks.

Four research camps have been built within the park – one in the middle of the range of each group (North, East, and South camp) and an additional quarantine camp in the northern area. Chimpanzees are followed daily by one to three researchers or assistants. Access of tourists and visitors is not possible to the park. For the Taï Chimpanzee Project (TCP), where this study has been carried out, only a small number of scientists (less than 10 in average) and local assistants (less than 30) have access to the camps and forest. Strict hygiene rules are in place to prevent transmission of any pathogens from people to animals [18], [24]. Persons who are about to approach the animals must be clinically healthy and have to go through 5 days quarantine after entering the park from outside. Face masks must be worn all the time in the presence of the chimpanzees and observers have to keep at least 7 m distance from them. Free defecation in the forest is not allowed; if necessary, faeces have to be collected immediately into plastic bags and transported to latrines at the camps (these are closed and not accessible to animals). Details of the hygienic protocol are available online [25].

The villages of Daobly, Ponan, Taï, Pauléola and Gouléako (with populations ranging from 1000 to 7000 inhabitants) are situated along the northern access road to TNP. People in the villages keep domestic animals such as dogs, cats, goats, cattle, poultry and pigs. The majority of assistants working in the park are residents of these villages; they come to work in the forest on four week rotations. There is no veterinarian operating in the region. According to questionnaire administered to participants of the study in the villages (M.P., unpublished data), dogs in the sampling area are not receiving antibiotics. People in the villages can acquire antibiotics from local hospitals or at local shops. Researchers working in the forest occasionally use doxycycline for malaria prophylaxis (for a period up to two months), however, treatment by beta-lactams or quinolones did not occur in the forest during the last five years.

### Sampling

Collection of faecal samples took place within and around the park from January 2012 to September 2012 (wildlife was sampled in January and February, people, dogs and cats from March to September). Chimpanzees of the three groups can be recognized individually, so individual faecal samples (n = 43) were taken. Faecal samples were collected into plastic bags immediately after defecation had been observed, without disturbing the natural behaviour [24]. A swab was made from the central part of the faeces. Additionally, fresh faeces of Diana monkey (*Cercopithecus diana,* n = 9), soothy mangabeys (*Cercocebus agilis,* n = 9), upper Guinea red colobus (*Pilocolobus badius,* n = 9) and King colobus (*Colobus polykomos*, n = 5) were sampled in a similar manner, but those cannot be considered as individual samples.

Within the quarantine camp, the latrine was sampled four times within a week interval (n = 4 in total) by a swab attached to a bamboo pole. Faeces were collected from nests of house mice (n = 4) in the camp. All swabs were preserved in Amies medium and kept refrigerated (the longest storage period was 8 weeks) until transported to the University of Veterinary and Pharmaceutical Sciences Brno, Czech Republic. In the villages samples were collected from 38 domestic dogs (12 in Daobly, 12 in Taï, 5 in Ponan, 9 in Pauléola) and 3 cats (all in Taï). Stool samples were also collected from 77 inhabitants of the villages (19 in Daobly, 39 in Ponan, 14 in Taï, 5 in Gouléako), stored in cryotubes in liquid nitrogen and transported on dry ice to Robert Koch Institute, Berlin, Germany and then stored at −80°C.

### Ethics statement

Sampling of humans was approved by the Ministère de la santé et de la lutte contre le SIDA, comité national d’éthique et de la recherche de la République de Côte d’Ivoire N°011-/MSHP/CNER-P. Before sampling, the aim of the study and the possibility to quit the study at any point, was explained individually in the local language. An individual study number was assigned to every participant to protect the privacy of the participant. Written informed consent was obtained from every study participant and sampling was performed according to the declaration of Helsinki. Sampling of domestic animals was done according to the Directive 86/609/EEC on the Protection of Animals Used for Experimental and Other Scientific Purposes. The permit for sampling of domestic animals was issued by LANADA, Laboratoire Nationale de la Pathologie Animale, Bingerville, CI. Sampling of wildlife in TNP was done non-invasively, according to permit issued by Office Ivoirien des Parcs et Réserves (OIPR), Abidjan, CI.

### Microbiological techniques

Each swab was plated in parallel on plain MacConkey agar (MCA) (Oxoid, UK), MCA with cefotaxime (2 mg/l) and MCA with ciprofloxacin (0.05 mg/l). The human samples were swabbed directly from the frozen material in vials and plated on MCA with cefotaxime and MCA with ciprofloxacin. The swabs originating from the park were plated additionally on MCA with cefoxitin (16 mg/l). One lactose-fermenting colony was isolated from each plate. All isolates grown on MCA with cefotaxime were tested by double.disc synergy test (DDST) for production of ESBL [26]. Selected isolates were tested by disc-diffusion method [26] for sensitivity to 12 antibiotics – ampicillin (10 µg), cephalotin (30 µg), amoxicillin-clavulanate (20+10 µg), ceftazidime (30 µg), trimethoprim-sulfamethoxazole (1.25+23.7 µg), sulphonamides compounds (300 µg), gentamicin (10 µg), nalidixic acid (30 µg), ciprofloxacin (5 µg), streptomycin (30 µg), tetracycline (30 µg) and chloramphenicol (30 µg). Isolates with resistance to all beta-lactams tested were additionally examined for sensitivity to aztreonam (30 µg), cefpodoxime (10 µg), cefepime (30 µg), imipenem (10 µg), meropenem (10 µg) and ertapenem (10 µg).

### Molecular typing

All isolates positive in DDST were tested by PCR for ESBL-encoding genes (*bla*_TEM_, *bla*_CTX-M_, *bla*_SHV_, *bla*_OXA_) as described previously [27]–[28]. Multiplex PCR [29] was used to search for genes of plasmid-mediated AmpC beta-lactamases in all isolates grown on plates containing cefoxitin and in isolates grown on media with cefotaxime but negative in the DDST. The PMQR genes (*aac(6′)-Ib-cr*, *qepA*, *qnrA*, *qnrB*, *qnrC*, *qnrD*, *qnrS* and *oqxAB*) were searched in isolates obtained on media with ciprofloxacin by PCRs as summarized elsewhere [30]. Forward primer psp2 was used in combination with reverse primers sc3, ds2 and ds3 [22] to investigate the flanking sequences of the *qnrB* genes. PMQR, ESBL and AmpC genes were sequenced to specify the type of the allele (EZ-seq service performed at Macrogen Europe). According to their resistance phenotype, isolates were tested for additional antibiotic resistance genes and integrons as described before [27].

All isolates with resistance were identified to species level by MALDI-TOF (matrix-assisted laser desorption/ionisation time-off-flight mass spectrometry; Microflex LT, Bruker Daltonics, Brehmen, Germany). *E. coli* isolates were tested for phylogroup by multiplex PCR [31]. All the ESBL-, AmpC- or PMQR- positive isolates were typed by XbaI pulsed-field gel electrophoresis (PFGE) [32]. The profiles obtained were analysed using the unweighed pair group method with arithmetic mean (UPGMA) in Bionumerics 6.6 software (Applied Maths, Sint-Martens-Latem, Belgium). Cluster analysis of the Dice similarity indices was conducted to generate a dendrogram describing the relationships among PFGE profiles.

### Transferability of resistance genes and plasmid characterization

Conjugation transfer of ESBL genes was made to plasmid-free, rifampin- and azid-resistant *E. coli* MT102RN and *Salmonella* Typhimurium SL5325 [33]. Plasmid DNA from ESBL-producing and PMQR-harbouring isolates was extracted by rapid alkaline method [34] and introduced into competent *E. coli* DH5α (Invitrogen, USA) by chemical transformation. Transformants were selected on LB agar (Difco, USA) supplemented with 2 mg/l cefotaxime or 0.05 mg/l ciprofloxacin, according to the genes of donor isolates. Transformants and transconjugants were tested for the number of plasmids gained and their size using S1-PFGE [35]. Single plasmid-harbouring and ESBL-producing transformants and transconjugants were screened for transferred resistance genes by PCR as described above. Plasmids extracted from these transformants and transconjugants by the Birnboim-Doly method were replicon typed [36]–[37] and their resistance genes were tested by PCR as described above [27], [30].

## Results

### Resistant isolates obtained from humans and domestic animals of the villages

The most common bacterial species obtained by cultivation of human- and dog- originating faecal samples on plates with ciprofloxacin and cefotaxime was *E. coli* (73/76; 96% isolates). All four phylogenetic groups were present; the most frequent group A (52/76; 68%) was followed in frequency by group B1 (14/76; 18%), D (12/76; 16%) and B2 (3/76; 0.4%). Based on the results of DDST (i.e. ESBL phenotype) and molecular methods, a total of 35 ESBL-producing *E. coli* isolates (Table 1) were obtained by cultivation of samples from dogs (12/38 dogs; 32%), cats (2/3 cats) and humans (21/77 humans; 27%). All the DDST-positive isolates produced the CTX-M-15 type beta-lactamase and were multiresistant; besides the resistance to beta-lactams, they showed resistance to other groups of antibiotics, including tetracyclines, sulphonamides, aminoglycosides, chloramphenicol and fluoroquinolones. Six of the ESBL-producing isolates have shown decreased sensitivity to ceftazidime. Among these, none was resistant to imipenem or meropenem, but all were uniformly resistant to cefepime, aztreonam and cefpodoxime. As revealed by transformation and conjugation experiments, the *bla*_CTX-M-15_ gene was localized on various conjugative plasmids ranging in size from 60 to 90 kb, of incompatibility group FIB or N, together with different combinations of other resistance genes (Table 2). Some plasmids were not typable by any of replicon-typing primers available. In total, transformation experiments were successful in 6 ESBL-producing isolates and conjugation was successful in 8 ESBL-producing isolates.

**Table 1:** Characteristics of ESBL-producing isolates from villages.

Locality	Source	Species, PG	Resistance genes	Resistance phenotype
Daobly	D 866 ctx	*E. coli*, A	*bla*_CTX-M-15_, *bla*_OXA1_,*strA, aadA5, tet*(B),*aac-(6′)-Ib-cr, sul2*	AmpCefNalCipTetSxtSptSul
	D 868 ctx	*E. coli*, D	*bla*_CTX-M-15_ (C), *bla*_OXA1_,*strA, aadA5, tet*(B),*aac-(6′)-Ib-cr, sul1*, *sul2*	AmpCefNalCipTetSxtChlSptSul
	D 878 ctx	*E. coli*, A	*bla*_CTX-M-15_, *strA,* *intI1* (*dfr1-sat-aadA1*),*sul2*	AmpCefNalSxtSul
	**D 879 ctx**	*E. coli*, A	*bla*_CTX-M-15_ (C),*bla*_TEM-1_, *strA*, *tet*(A), *sul2*	AmpCefTetSxtSptSul
	H 260 ctx	*E. coli*, A	*bla*_CTX-M-15_, *tet*(A)	AmpCefTet
	H 267 ctx	*E. coli*, A	*bla*_CTX-M-15_, *tet*(A),*sul2, aadA2*	AmpCefNalGenTetSxtSptSul
	**H 268** **ctx**	*E. coli*, A	*bla*_CTX-M-15_ (T, C),*bla*_TEM-1_, *tet*(B), *sul2*	AmpCefCazTetSxtSptSul
	H 271 ctx	*E. coli*, B2	*bla*_CTX-M-15_, *bla*_TEM-1_,*strA*, *tet*(A), *sul1*,*sul, aadA5*	AmpCefCazNalCipGenTetSxtSptSul
	H 285 ctx	*E. coli*, B1	*bla*_CTX-M-15_, *strA*,*tet*(A), *sul2, aadA2*	AmpCefNalTetSxtSptSul
	H 298 ctx	*E. coli*, D	*bla*_CTX-M-15_, *strA,* *tet*(A), *aac-(6′)-Ib-cr,* *aac(3)II, sul2*	AmpCefAmcNalCipGenTetSxtSptSul
	H 324 ctx	*E. coli*, B1	*bla*_CTX-M-15_,*tet*(A), *sul2*	AmpCefTetSxtSptSul
	**H 332** **ctx**	*E. coli*, B1	*bla*_CTX-M-15_ (T),*bla*_TEM-1_, *tet*(A), *sul2*	AmpCefTetSxtSptSul
Ponan	H 106 ctx	*E. coli*, A	*bla*_CTX-M-15_, *strA*,*tet*(B), *aac-(6′)-Ib-cr,* *sul1*, *sul2, aadA5*	AmpCefCazNalCipTetSxtSptSul
	H 110 ctx	*E. coli*, A	*bla*_CTX-M-15_, *bla*_TEM-1_,*strA*, *tet*(A), *sul2, aadA2*	AmpCefTetSxtSptSul
	H 115 ctx	*E. coli*, A	*bla*_CTX-M-15_, *bla*_TEM-1_,*strA*, *tet*(A), *sul2*,*sul3, aadA2*	AmpCefCpdTetSxtSptSul
	H 127 ctx	*E. coli*, D	*bla*_CTX-M-15_, *bla*_TEM-1,_ *aac(3)II, aadA2, aadA5*	AmpCefCazAmcNalCipGenTetSxtSptSul
	H 149 ctx	*E. coli*, D	*bla*_CTX-M-15_, *strA, sul2*, *sul3*	AmpCefCazNalTetSxtChlSptSul
	H 154 ctx	*E. coli*, A	*bla*_CTX-M-15_, *tet*(B), *aadA5*	AmpCefAmcNalCipTetSxtChlSptSul
	H 167 ctx	*E. coli*, A	*bla*_CTX-M-15_, *tet*(A),*sul2, aadA2*	AmpCefNalTetSxtSptSul
Taï	D 856 ctx	*E. coli*, A	*bla*_CTX-M-15_, *bla*_TEM-1_,*strA*, *tet*(A)	AmpCefCipTetSxtSptSul
	**D 860** **ctx**	*E. coli*, A	*bla*_CTX-M-15_, *bla*_TEM-1_,*strA*, *tet*(B), *sul2*	AmpCefTetSxtSptSul
	**D 872** **ctx**	*E. coli*, A	*bla*_CTX-M-15_ (T),*bla*_TEM-1_, sul2	AmpCefTetSxtSptSul
	C 881 ctx	*E. coli*, A	*bla*_CTX-M-15_ (C),*bla*_TEM-1_, *strA*, *tet*(A), *sul2*	AmpCefTetSxtChlSptSul
	C 882 ctx	*E. coli*, B1	*bla*_CTX-M-15_, *strA*,*tet*(A), *qnrS1*	AmpCefNalCipTetSxtSptSul
	D 884 ctx	*E. coli*, B1	*bla*_CTX-M-15,_ bla_OXA1_,*bla*_TEM-1_, *strA*, *intI2*(*dfr1-sat-aadA1*),*aac-(6′)-Ib-cr, sul2*	AmpCefNalCipTetSxtSptSul
	**H 215** **ctx**	*E. coli*, B1	*bla*_CTX-M-15_ (T),*bla*_TEM-1_, *strA*,*tet*(A), *aadA2*	AmpCefTetSxtSptSul
	H 217 ctx	*E. coli*, A	*bla*_CTX-M-15_ *strA*,*tet*(A), *aadA5*	AmpCefNalTetSxtSptSul
	H 234 ctx	*E. coli*, A	*bla*_CTX-M-15_, *strA*,*tet*(A), *aadA5*	AmpCefTetSxtSptSul
	H 241 ctx	*E. coli,* A	bla_OXA1_, *bla*_TEM-1_	AmpCefCazAmcNalCipGenTetSxtChlSptSul
	**H 244** **ctx**	*E. coli*, B1	*bla*_CTX-M-15_ (C),*strA*, *tet*(A), *aadA2*	AmpCefNalTetSxtSptSul
Pauléola	**D 855** **ctx**	*E. coli*, A	*bla*_CTX-M-15_ (C),*bla*_TEM-1_, *strA*, *tet*(B), *sul2*	AmpCefNalCipGenTetSxtSptSul
	**D 858** **ctx**	*E. coli*, A	*bla*_CTX-M-15_ (C),bla_OXA1_, *strA*, *aadA5*,*tet*(B), *aac-(6′)-Ib-cr, sul2*	AmpCefNalCipTetSxtSptSul
	D 859 ctx	*E. coli*, A	*bla*_CTX-M-15_,*bla*_TEM-1_, *strA*,*tet*(A), *sul2*	AmpCefTetSxtChlSptSul
	D 886 ctx	*E. coli*, B1	*bla*_CTX-M-15_, *strA,* *aadA5, intI2*(*dfr1-sat1*), *sul2*	AmpCefNalCipGenTetSxtChlSptSul
Gouléako	**H 344** **ctx**	*E. coli*, B1	*bla*_CTX-M-15_ (C),*bla*_TEM-1_, *tet*(A),*aac(3)II, sul2, aadA2*	AmpCefTetSxtSptSul

All isolates were obtained on MCA-cefotaxime.

“D” – isolate from dog, “H” – isolates from human, “C” – isolate from cat. “T” in brackets means that the resistance gene was successfully transformed into competent cells, “C” in brackets means that the gene was conjugated. Plasmids were isolated and characterized from isolates in bold font. See Table 2 for plasmid characteristics.

PG = phylogroup, Amp = ampicillin, Cef = cephalotin, Caz = ceftazidime, Amc = amoxycilin-clavulanate, Nal = nalidixic acid, Cip = ciprofloxacin, Tet = tetracycline, Sxt = trimethoprim-sulfamethoxazole, Spt = streptomycin, Sul = sulfonamides compounds, Gen = gentamicin, Chl = chloramphenicol, Cpd = cefpodoxime.

**Table 2:** Characteristics of plasmids obtained by transformation.

Isolate ID	Donor isolatespecies	Locality ofdonor isolate	Incgroup	Size (kb)	Resistance genesco-transferredon the plasmid
H 115 cip	*E. coli*	Ponan	NT	35	*qnrS1*
D 854 cip	*E. coli*	Taï	FIB	75	*qnrS1, tet*(A), *sul2, strA, bla*_TEM-1_
D 855 cip	*E. coli*	Pauléola	FIB	75	*qnrS1, tet*(A), *sul2, strA, bla*_TEM-1_
D 866 cip	*E. coli*	Daobly	NT	33	*qnrS1, sul3*
D 867 cip	*E. cloacae*	Daobly	R	75	*qnrS1, sul2, strA*
D 871 cip	*E. coli*	Taï	NT	40	*qnrS1, strA, bla*_TEM-1_
D 877 cip	*E. coli*	Pauléola	NT	35	*qnrS1, tet*(A)
D 879 cip	*E. coli*	Daobly	NT	90	*qnrS1, bla*_TEM-1_
H 215 ctx	*E. coli*	Taï	NT	70	*bla*_CTX-M-15_, *bla*_TEM-1_, *tet*(A), *sul2*, *strA*
H 244 ctx	*E. coli*	Taï	NT	70	*bla*_CTX-M-15_, *sul2, strA*
H 268 ctx	*E. coli*	Daobly	NT	70	*bla*_CTX-M-15_, *sul2*
H 332 ctx	*E. coli*	Daobly	NT	70	*bla*_CTX-M-15_, *bla*_TEM-1_, *tet*(A), *sul2*, *strA*
H 344 ctx	*E. coli*	Gouléako	NT	65	*bla*_CTX-M-15_, *tet*(A)
D 859 ctx	*E. coli*	Pauléola	N	75	*qnrS1*, *bla*_TEM-1_, *tet*(A), *sul2*, *strA*
D 860 ctx	*E. coli*	Taï	NT	60	*bla*_CTX-M-15_, *bla*_TEM-1_, *sul2*, *strA*
D 872 ctx	*E. coli*	Taï	FIB	75	*bla*_CTX-M-15_, *tet*(A), *sul2*, *strA*
D 879 ctx	*E. coli*	Daobly	FIB	90	*bla*_CTX-M-15_, *bla*_TEM-1_, *te*t(A), *sul2*, *strA*

“NT”–not typable. Note: transconjugants obtained in this study contained more than one plasmid and therefore were not used for plasmid characterization.

By cultivation on MCA with ciprofloxacin, a total of 44 PMQR isolates was obtained from samples collected in the villages (Table 3). Of these, 14 dog-originating isolates of *E. coli* and 20 human-originating isolates of *E. coli* possessed the gene *qnrS1*. The gene *qnrB* was found in *E. coli* from three people and one *C. freundii* isolate from a dog, the *aac-(6′)-Ib-cr* was detected in two human-originating *E. coli* isolates and one human-originating *K. pneumoniae*. The *aac-(6′)-Ib-cr* gene was also present in four of the ESBL-producing isolates. One *E. coli* isolate had the *oqxAB* efflux pump. Similarly to the ESBL-producing isolates, the PMQR isolates were multiresistant; three to eight of the antibiotics tested have shown decreased sensitivity in the disc-diffusion test.

**Table 3:** Characteristics of isolates with PMQR genes.

Locality	Source, ID	Species, PG	Resistance genes	Resistance phenotype
Daobly	D 862 cip	*E. coli*, A	*qnrS1*, *tet*(A),*strA*, *bla*_TEM-1,_ *sul2*	AmpTetSxtSulSpt
	D 863 cip	*E. coli*, A	*qnrS1*, *tet*(A),*bla*_TEM-1,_ *sul2*	AmpTetSxtSulSpt
	D 864 cip	*E. coli*, A	*qnrS1*, *tet*(A),*strA*	AmpTetSxtSulSpt
	**D 866** **cip**	*E. coli*, A	*qnrS1*(T),*bla*_TEM-1,_ *sul3*	AmpCefTetSxt
	**D 867** **cip**	*E. cloacae*	*qnrS1* (T), *tet*(A),*strA*, *bla*_TEM-1,_ *sul2*	AmpCefAmcTetSxtSulSpt
	D 878 cip	*E. coli*, D	*qnrS1*, *tet*(A),*strA*, *bla*_TEM-1,_ *sul2*	AmpCefAmcTetSxtSulSpt
	**D 879** **cip**	*E. coli*, A	*qnrS1* (T), *bla*_TEM-1_	AmpSulSxt
	D 883 cip	*E. coli*, A	*qnrS1*, *tet*(A),*strA, sul2*	AmpTetSulSxtSpt
	H 270 cip	*K. pneumoniae*	*aac-(6′)-Ib-cr*, *oqxB*,*tet*(A), *aadA5*	AmpCefTet
	H 298 cip	*E. coli*, D	*aac-(6′)-Ib-cr*,*tet*(A), *tet*(B)	AmpNalCipGenTetSxtSul
	H 285 cip	*E. coli*, A	*qnrS1*, *tet*(A)	AmpTetSxtSul
	H 295 cip	*E. coli*, B1	*qnrS1*, *tet*(A)	AmpTetSxtSulSpt
	H 324 cip	*E. coli*, A	*qnrB1*, *tet*(A)	SulSxtTet
	H 336 cip	*E. coli*, A	*qnrS1, tet*(A),*intI1* (*dfrA1-aadA1*)	AmpTetSulSxt
Ponan	H 103 cip	*E. coli*, A	*qnrS1*, *oqxA*, *tet*(A),*intI1* (*dfrA1-aadA1*),*aadA2*	AmpTetSxtSul
	H 109 cip	*E. coli*, A	*qnrS1, sul1*	AmpNalTetSxtSul
	H 110 cip	*E. coli*, A	*qnrS1*, *tet*(A),*strA, intI2* (*dfrA1-sat-aadA1),* *sul2, aadA2*	AmpCefTetSxtSul
	H 111 cip	*E. coli*, A	*aac-(6′)-Ib-cr*,*tet*(A), *strA, sul1, sul2*	AmpNalCipTetSxtSul
	H 112 cip	*E. coli*, B2	*qnrS1*, *oqxA*	AmpTetNal
	**H 115** **cip**	*E. coli*, A	*qnrS1* (T, C), *tet*(A)	AmpSulTet
	H 120 cip	*E. coli*, A	*qnrS1*, *tet*(A)	AmpSulTetChl
	H 125 cip	*E. coli*, A	*oqxAB*, *aac-(6′)-Ib-cr*,*tet*(A), sul1	SxtSulTetNalCip
	H 127 cip	*E. coli*, D	*qnrB1*, *aac-(6′)-Ib-cr,* *intI1* (*aadA1*), *aac(3)II, aadA2*	AmpCefSulSxtChlGen
	H 151 cip	*E. coli*, A	*qnrS1*, *tet*(A)	AmpSulTetSxt
	H 158 cip	*E. coli*, A	*qnrS1*, *tet*(A)	AmpSulTetSxt
	H 159 cip	*E. coli*, A	*qnrB19*, *tet*(A), *strA, sul3*	AmpSulTetSxt
	H 167 cip	*E. coli*, A	*qnrS1*, *oqxA*, *tet*(A),*strA, intI2*(*dfrA1-sat-aadA1), sul2*	AmpTetSxtSulSptCfa
Taï	H 212 cip	*E. coli*, A	*qnrS1*, *tet*(A)	AmpTetSxtSul
	H 217 cip	*E. coli*, A	*qnrS1*, *tet*(A)	AmpTetSxtSul
	H 220 cip	*E coli*, A	*qnrS1*, *tet*(A),*strA, sul2, aadA2*	AmpCipTetSxtSulSpt
	H 224 cip	*E. coli*, B1	*qnrS1*, *tet*(A), *sul2*	AmpTetSxtSulSpt
	H 227 cip	*E. coli*, A	*qnrS1*, *tet*(A), *strA, sul2*	AmpTetSxtSul
	H 228 cip	*E. coli*, A	*qnrS1*, *tet*(A)	AmpTetSxtSulSpt
	H 234 cip	*E. coli*, A	*qnrS1*, *tet*(A), *sul2, aadA2*	AmpTetSxtSulChlSpt
	**D 854** **cip**	*E. coli*, B1	*qnrS1* (T), *tet*(A), *strA*, *bla*_TEM-1_, *sul2*	AmpCefTetSxtSulSpt
	**D 871** **cip**	*E. coli*, A	*qnrS1* (T), *tet*(A),*strA*, *bla*_TEM-1_	AmpCefCipTetSulSxtSpt
	D 872 **cip**	*E. coli*, A	*qnrS1*, *tet*(A), *strA*,*bla*_TEM-1_, *sul2*	AmpNalCipTetSulSxtSpt
Gouléako	H 344 cip	*E. coli*, A	*qnrS1*, *tet*(A), *strA,* *sul2, aadA5*	AmpTetSxtSul
	H 346 cip	*E. coli*, A	*aac-(6′)-Ib-cr*, *tet*(B),*strA, sul2*	AmpNalCipGenTetSulSxtSpt
Pauléola	**D 855** **cip**	*E. coli*, B1	*qnrS1* (T), *tet*(A),*strA*, *bla*_TEM-1_, sul2	AmpCefTetSxtSulSpt
	D 858 cip	*E. coli*, B1	*qepA*, *tet*(B),*bla*_TEM-1_, *intI1* (*dfr2d*), *sul1*	AmpCefAmcNalCipTetSxtSul
	**D 859** **cip**	*E. coli*, A	*qnrS1* (T), *tet*(A),*strA*, *bla*_TEM-1,_ *sul2*	AmpTetSxtSulSpt
	D 869 cip	*C. freundii*	*qnrB28*	Amp
	**D 877** **cip**	*E. coli*, D	*qnrS1* (T), *tet*(A), *bla*_TEM-1_	AmpNalTetSul

Isolates were obtained on MCA-ciprofloxacin.

For legend see table 1.

**Figure 1:**
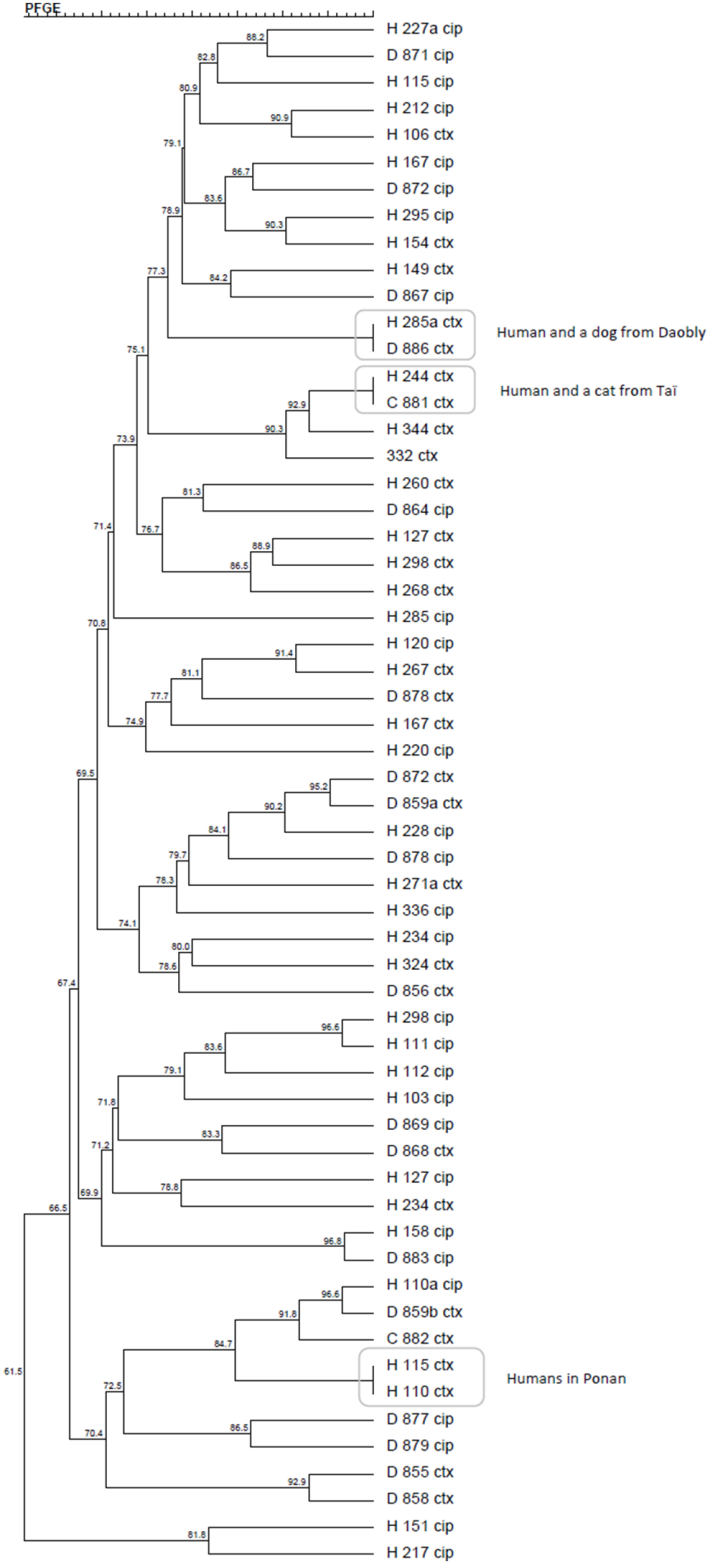
Dendrogram of resistant *E. coli* isolates’ PFGE profiles. Generated by cluster analysis of the Dice similarity indices in the BioNumerics fingerprinting software (optimization 1%, band matching tolerance 1%, tolerance change 1%). Isolates marked with “cip” were obtained by cultivation on ciprofloxacin and harbored PMQR genes, isolates with “ctx” represent the CTX-M-15 producing *E. coli*. “H” isolate from human, “D” isolate from dog, “C” isolate from cat.

According to the PFGE analysis, the ESBL- and PMQR- resistant isolates grouped into 15 clusters of >80% similarity irrespective of their host (dogs versus humans) and sampling site (village). No clone seemed to be characteristic of a given locality. Isolates with identical PFGE profile were detected in humans and animals living in the same villages (Figure 1).

### Resistant isolates found in NHPs and research camps in the forest

No lactose-fermenting colonies were obtained by cultivation of the 76 samples obtained within TNP (NHPs, mice, the latrine in the research camp) on plates with cefotaxime. A total of 25 lactose-fermenting colonies were obtained on MCA with ciprofloxacin (Table 4). Among those, four isolates originating from chimpanzees and three originating from monkeys were identified as *Citrobacter freundii* and carried variants of *qnrB,* one chimpanzee harboured a *qnrB13-*producing *Klebsiella pneumoniae.* A *qnrB-*harbouring *C. freundii* was found also in one house mouse in the North camp. The allele *qnrB13* was found exclusively in chimpanzees, *qnrB9* occurred only in monkeys and *qnrB28* was detected in chimpanzees, monkeys, house mouse and also in a dog living in Pauléola village. Besides the *qnrB*-harboring isolates, the cultivation on MCA with ciprofloxacin generated five wildlife-originating *K. pneumoniae* with *oqxA* gene (not shown in tables) and one multiresistant *E. coli* originating from the camp latrine (Table 4).

**Table 4:** Isolates with PMQR or AmpC genes originating from TNP (wildlife and camps).

Territory	sampleorigin	bacterialspecies	resistancegenes	resistancephenotype
North	latrine, quarantinecamp 789 cip	*E. coli*	*oqxA*	AmpNalCipTetSxtSulChlSpt
	house mouse 785 cip	*C. freundii*	*qnrB28*	sensitive
	house mouse 786 fox	*E. asburiae*	*bla_EBC_*	AmpCef
	King colobus 794 cip	*C. freundii*	*qnrB28, oqxA*	AmpAmc
	Diana monkey 792 cip	*C. freundii*	*qnrB9*	sensitive
	upper Guineared colobus 803 cip	*C. freundii*	*qnrB9* (C), *oqxA*	Amc
	soothy mangabey796 fox	*C. freundii*	*bla_EBC_*	AmpCef
	western chimpanzee820 cip	*C. freundii*	*qnrB13, oqxA*	AmpAmc
	western chimpanzee833 cip	*K. pneumoniae*	*qnrB13* (C), *oqxA*	AmcCip
	western chimpanzee809 fox	*E. asburiae*	*bla_CMY_*	AmpCef
	western chimpanzee809 cip	*C. freundii*	*qnrB13*	AmpAmc
South	western chimpanzee826 fox	*E. asburiae*	*bla_ACT_, bla_DHA_, bla_CMY_*	AmpCef
East	western chimpanzee846 cip	*C. freundii*	*qnrB28*	sensitive
	western chimpanzee849 cip	*C. freundii*	*qnrB13*	sensitive

Isolates with suffix “cip” were obtained on MCA-ciprofloxacin, isolates with suffix “fox” were obtained on MCA-cefoxitin.

Cefoxitin-resistant isolates of *Enterobacter asburiae* or *C. freundii* were obtained by cultivation of the chimpanzee, mouse and mangabey samples on MCA-cefoxitin and showed to harbour *bla*_DHA_, *bla*_CMY_, *bla*_EBC_ and *bla*_ACT_ genes (Table 4). Cultivability of *E. coli* from the wildlife-originating swabs was checked by their growth on MCA without antibiotics; *E. coli* was successfully obtained from all samples. Of the non-selectively cultivated isolates, two, originating from chimpanzees (821 and 842 – North and East group individuals, respectively), have shown resistance to multiple antibiotics (phenotypes AmpCefAmcSulSxt and AmpSulSpt; not shown in tables). Within other isolates obtained on the non-selective medium, five had decreased sensitivity to ampicillin, while the rest were sensitive to all 12 antibiotics.

## Discussion

### Resistance found in the villages

Multiresistant *E. coli* with the CTX-M-15 beta-lactamase were detected in 32% of people and 27% of dogs living in the investigated villages. Similar prevalence has been recently recorded in other rural communities in Africa [38]–[41]. CTX-M actually represents a pandemic type of extended-spectrum beta-lactamase with faecal carriage prevalence reaching up to 66% [42]. The particular success of the allele *bla*_CTX-M-15_ in human population has not been satisfactorily clarified, but its selection by use of ceftazidime and other third-generation cephalosporins is the very likely initial step [43]. In Africa, the use of third-generation cefalosporins is often warranted as a second-line alternative to treat multiresistant cholera, pneumonia, typhoid fever and other serious but frequent infections [44]. CTX-M-15 is known to occur on conjugative multiresistance-encoding plasmids, which was the case also in our study. Such plasmids can facilitate the process of co-selection [45] so that the beta-lactamase can be spread even under selective pressure of more common and cheaper antibiotics like sulfonamides, tetracyclines or aminoglycosides. Frequent need of antimicrobial treatment, together with poor sanitary conditions and warm climate, make ideal conditions for a widespread ESBL colonisation of people and animals in rural communities [13]. Although dogs are not directly subjected to the selective pressure exerted by antibiotic therapy, they often scavenge for food and water in human refuse (including the “free defecation zones”; [46]) and thus get easily colonized by human-originating bacteria from the environment.

The PMQR isolates, obtained by cultivation on ciprofloxacin, were dominated by *qnrS1-*harboring *E. coli*. This gene probably originates from *Salmonella* sp. and its spread to other Enterobacteriaceae is associated with the increased use of fluoroquinolones [12]. The genes *qnrB* or *oqxAB* represented a minority within human-originating PMQR. Some of the PMQR isolates were sensitive to clinical levels of fluoroquinolones, as shown by the disc-diffusion method. This is a commonly observed phenomenon and does not decrease the epidemiological significance of PMQR genes in a given ecosystem [11]. As shown by PFGE-based dendrogram (Figure 1), the genes *bla*_CTX-M-15_ and *qnrS1* were not spread across the community by one or several clones, but were present in many different clonally unrelated isolates. This fact highlights the epidemiological importance of plasmid-mediated resistance genes, which can cause fast spreading of resistance in a particular area. The PFGE analysis revealed another interesting phenomenon: although dogs and people were sampled three months apart, isolates with identical PFGE profile were revealed in dogs and people living in the same place or in neighbouring villages (Figure 1). This finding corroborates the general presumption that, once established in an ecosystem, resistant bacterial clones can persist for a long time.

All four phylogroups have been detected among the ESBL and PMQR *E. coli* isolates obtained from people and domestic carnivores. While closely related groups A and B1 are usually regarded as non-pathogenic, more virulence factors are found in *E.coli* isolates of groups B2 and D [47]. Determination of pathogenicity of the investigated samples would undoubtedly be interesting, however it was not possible within the scope this study. The predominance of phylogroup A is in accordance with findings of Carlos et al. [48], who concluded that group A is more prevalent in omnivorous hosts, while group B1 is typical for herbivorous hosts.

### Resistance found in the forest

In TNP the overall prevalence of resistant bacteria in chimpanzees and monkeys was very low and most of it actually could have been attributed to the naturally evolving resistome, especially because the monkeys have never been treated with antibiotics. The only *E. coli* isolates with resistance to more than two antibiotics (however with no ESBL or PMQR genes) within the park were found in two chimpanzees from northern and eastern group, however, none was found in the southern group, that had been recently treated for the respiratory outbreak by benzylpenicillin. Other isolates with resistance were *Citrobacter* or *Enterobacter* isolates with AmpC or *qnr* genes. It should be pointed out that Enterobacters are considered the natural reservoir of AmpC alleles, which are typically localized on their chromosomes and make part of their intrinsic resistance [10]. As such, the AmpC-harbouring *Enterobacter* isolated from wildlife animals can be considered a part of their innate microflora. Likewise, *Citrobacter* sp. is supposed to play the role of natural reservoir and “evolutionary cradle” of *qnrB* alleles [22]. These genes occur on chromosomes (their original function is not known) from where they are often mobilized by insertion sequences to plasmids and later can disseminate to other bacterial species, including human pathogens [49]. The *qnrB*–carrying plasmids are often conjugative, facilitating such spread [22].

### No evidence of MDR-Enterobacteriace transmission from humans to NHPs

The hygiene rules employed by TCP include changing of clothes, washing of hands and boots at hygiene barriers before leaving the camps and entering the forest, prohibition of any defecation in the forest (stool is carried back to camps) and leaving any food or other remains in the forest. This study gave an opportunity to test the efficiency of the rules in place. Despite the presence of multiresistant *E. coli* with the CTX-M-15 beta-lactamase or/and *qnrS1* plasmid-mediated quinolone resistance genes in the villages around TNP, no such isolate was detected in NHPs living in the park. Considering the fact that all assistants working in the forest are residents in the sampled villages and most of the food supplies delivered to the research camps also originate in these villages, our comparison indicates that the hygienic rules employed in TNP are efficient to protect Taï NHPs against acquisition of human-originating MDR-Enterobacteriaceae. Absence of evidence however, is not evidence of absence and more longitudinal data are necessary to test this case further. It is also worth considering that the microbiota might be host-adapted, so exposure might occur without following colonization. Similar studies were conducted in primates living in Lopé National Park, Gabon [14] and Bwindi Impenetrable National Park, Uganda [16]. Direct transmission of resistant bacteria from human to great apes has not been demonstrated in either case. However, none of these studies investigated the resistance carrying plasmids and therefore they might disregard the aspect of horizontal gene flow between microbial populations.

### Possible transfer of bacteria from wildlife to people

We found *qnrB28-*harboring *Citrobacter freundii* in a chimpanzee, a King colobus, a research camp-dwelling house mouse and also in a dog living in Pauléola, a village very close to the entry to the park. A consumption chain might exist among these animals, because dogs are often used for hunting and are in contact with remnants of meat and organs of wildlife through bushmeat hunting. Handling and consumption of bushmeat represents a risk of transmission of wildlife-originating bacteria (including isolates with naturally evolving resistance genes) also to people, similarly to what was shown before for various retroviruses [50]–[51].

### Conclusion and Study Limitations

In conclusion, we found no significant overlap between the resistant Enterobacteriaceae in people and animals living around the park and wildlife dwelling in the park. While multiresistant *E. coli* with ESBL genes carried on conjugative plasmids were detected among people and dogs, no such bacteria were found in wildlife within the park. PMQR genes found outside the park were different from those detected in TNP-dwelling wildlife, the only exception represented by *qnrB28* in *Citrobacter freundii.* This study was limited in time and size of the sample set; further sampling and investigation of other resistance genes groups would be necessary to determine the extent of overlap between the commensal microbiota of people and wildlife in the area.
